# Antioxidant and C5a-blocking strategy for hepatic ischemia–reperfusion injury repair

**DOI:** 10.1186/s12951-021-00858-9

**Published:** 2021-04-15

**Authors:** Xiaobing Zhang, Jiajia Hu, Kaelyn V. Becker, Jonathan W. Engle, Dalong Ni, Weibo Cai, Dong Wu, Shuping Qu

**Affiliations:** 1 Department of Hepatobiliary Surgery, Eastern Hepatobiliary Surgery Hospital, Second Military Medical University, Shanghai, 200438 People’s Republic of China; 2 Department of Nuclear Medicine, Ruijin Hospital, Shanghai Jiaotong University School of Medicine, Shanghai, 200025 People’s Republic of China; 3 Departments of Radiology and Medical Physics, University of Wisconsin–Madison, Madison, WI 53705 USA

**Keywords:** Hepatic ischemia–reperfusion injury, Nanoantioxidants, C5a, Nanoceria, Aptamer

## Abstract

**Background:**

Nonspecific liver uptake of nanomaterials after intravenous injection has hindered nanomedicine for clinical translation. However, nanomaterials’ propensity for liver distribution might enable their use in hepatic ischemia–reperfusion injury (IRI) repair. During hepatic IRI, reactive oxygen species (ROS) are generated and the fifth component of complement (C5a) is activated. In addition, C5a is confirmed to exacerbate the vicious cycle of oxidative stress and inflammatory damage. For these reasons, we have investigated the development of nanomaterials with liver uptake to scavenge ROS and block C5a for hepatic IRI repair.

**Results:**

To achieve this goal, a traditional nanoantioxidant of nanoceria was surface conjugated with the anti-C5a aptamers (Ceria@Apt) to scavenge the ROS and reduce C5a-mediated inflammation. High uptake of Ceria@Apt in the liver was confirmed by preclinical positron emission tomography (PET) imaging. The clinical symptoms of hepatic IRI were effectively alleviated by Ceria@Apt with ROS scavenging and C5a blocking in mice model. The released pro-inflammatory cytokines were significantly reduced, and subsequent inflammatory reaction involved in the liver was inhibited.

**Conclusions:**

The synthesized Ceria@Apt has great potential of medical application in hepatic IRI repair, which could also be applied for other ischemic-related diseases.

**Graphic abstract:**

**Supplementary Information:**

The online version contains supplementary material available at 10.1186/s12951-021-00858-9.

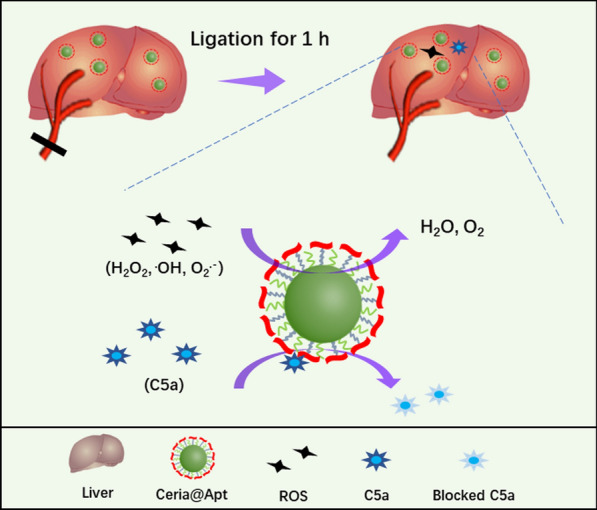

## Introduction

Nanotechnology’s unique physiochemical properties portend a variety of useful medical applications [1–4]. Several multifunctional nanomaterials, including organic nanoparticles (NPs), inorganic NPs, and organic/inorganic hybrid NPs, have been synthesized and applied for multimodal imaging, cancer therapy, tissue regeneration, and other applicaitons [5–14]. However, nonspecific uptake of nanomaterials by mononuclear phagocyte systems (MPS, e.g., liver, spleen) after intravenous injection is the biggest hurdle to human use [15, 16]. To overcome this obstacle in translational nanomedicine, researchers pursued understanding the delivery mechanism of NPs in MPS and engineering of nanomaterials to decrease MPS uptake by size control, surface modification, and surface potential regulation [17–20]. In addition, researchers also utilized NPs with preferential liver uptake for the treatment of hepatic disease, such as liver fibrosis [21–26], hepatic ischemia–reperfusion injury (IRI) [27–32], hepatic cancer [33–35], etc. Due to preferred liver accumulations, hepatic diseases have been one of the most promising research directions in translation of nanomedicine. [36–38].

Hepatic IRI by reactive oxygen species (ROS) generated upon re-oxygenation has been regarded as a major cause of hepatic dysfunction and failure after surgery [39–41]. Nano-antioxidants with excellent ROS scavenging capability include nanoceria [28], carbohydrates [27], and bilirubin nanoparticles [29] have been reported to alleviate hepatic IRI. We recently revealed the process of hepatic IRI repair by nanoceria, including ROS scavenging, inactivation of Kupffer cells, and inhibition of the recruitment and infiltration of neutrophils [28]. Finally, the subsequent inflammatory response involved was greatly inhibited. However, the ROS scavenging of nanoceria, especially superoxide radical, depends on the valence of the cerium ion, and a mild damage was still found at 12 h after treatment [28]. As a pro-inflammatory polypeptide, the fifth component of complement (C5a) is a potent chemotactic factor that can improve superoxide radical formation during ischemic process [42–44]. It has been confirmed that C5a plays important roles in exacerbating the vicious cycle of oxidative stress and inflammatory damage. Therefore, antioxidant treatment and C5a-blocking strategies could be beneficial in the management of ischemic IRI [45, 46], but they have not been developed.

**Fig. 1:**
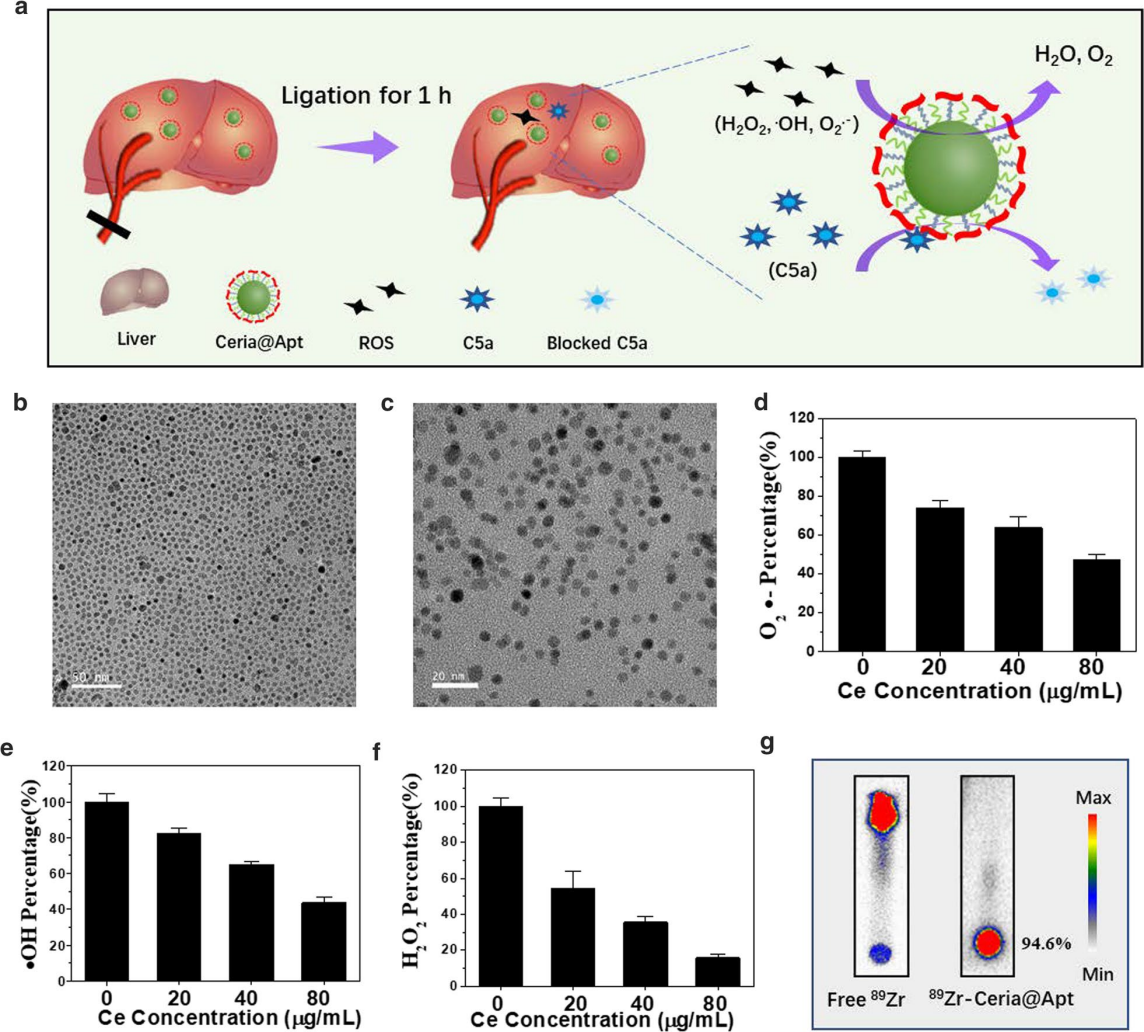
Characterization of Ceria@Apt. **a** Schematic illustration of Ceria@Apt for hepatic IRI repair. TEM images of nanoceria **b** before and **c** after surface modification. **d** O^2•−^, **e** •OH, and **f** H_2_O_2_ scavenging activity of Ceria@Apt (n = 3, means ± s.d). **g** Autoradiographic image of thin layer chromatography (TLC) plates after radiolabeling ^89^Zr to Ceria@Apt. Free ^89^Zr-oxalate was used as a control

The anti-C5a aptamer is a short, single-stranded oligonucleotide with high affinity and specificity for C5a, but it is alive to nuclease degradation and unstable in serum when delivered alone. Herein, the anti-C5a aptamers was modified on the surface of nanoceria (Ceria@Apt) to scavenge the ROS and reduce C5a-mediated inflammatory and damage. As illustrated in Fig. 1a, after accumulation in the liver, the Ceria@Apt could scavenge ROS by nanoceria and block C5a by anti-C5a aptamer to reduce the involved inflammatory and alleviate hepatic IRI. The strategy of uniting nano-antioxidants and anti-C5a for the repair of IRI not only provides the supplement of anti-oxidative therapy for hepatic IRI repair but also enriches the application of nanomedicine in other ischemic diseases.

## Experimental section

### Materials

Cerium (III) nitrate hexahydrate (Ce(NO3)3·6H_2_O, 99%), oleylamine (70%), 1-octadecene (ODE, 90%), cyclohexane (99.5%), and chloroform were purchased from Sigma-Aldrich. DSPE-PEG_5K_ and DSPE-PEG_5K_-NH_2_ was purchased from Creative PEG works. Anti-C5a oligonucleotides were purchased from Integrated DNA Technologies Inc. (www.IDTDNA.com). All reagents were used without any purification.

### Synthesis of Ceria@Apt

Firstly, nanoceria was synthesized using a previously established method, followed by surface modification with biocompatible DSPE-PEG_5k_ and DSPE-PEG_5k_-NH_2_ (Ratio, 5:1) through a hydrophilic and hydrophobic interaction [28]. Since anti-C5a aptamer is negative, it is easy to absorb on the surface of PEGylated ceria with strong positive charge due to the -NH_2_ group.

### Radiolabeling of Ceria@Apt

The Ceria@Apt was radiolabeled with ^89^Zr produced using the ^89^Y(p,n)^89^Zr nuclear reaction to evaluate its in vivo biodistribution through PET imaging. Briefly, 200 μL of nanoceria dispersed in HEPES buffer was mixed with1 mCi (or 37 MBq) of ^89^Zr-oxalate and the final pH value of the solution was adjusted to 7–8 by adding 1 M Na_2_CO_3_. After shaking for 2 h at 55 °C, ^89^Zr-Ceria@Apt could be easily collected from a PD-10 column with PBS as the mobile phase. Thin layer chromatography (TLC) with subsequent autoradiography (Packard Storage Phosphor) were used to monitor and quantify the radiolabeling yield. EDTA (0.5 M) in deionized water was used as the developing solvent.

### Characterization

The concentration of cerium was measured using ICP-OES (Agilent 725, Agilent Technologies). The transmission electron microscope (TEM) images of nanoceria were performed using JEM-2100. The confocal laser scanning microscopy (CLSM) images were recorded from the A1R microscope (Nikon Co.).

### ROS scavenging activity assay

Three main ROS, H_2_O_2_, ^•^OH, and O_2_^−^ were used to evaluate the ROS scavenging capability of Ceria@Apt. The H_2_O_2_ quenching activity was conducted with the Amplex® red hydrogen peroxide/peroxidase assay kit (Molecular Probes, Inc., USA). The ^•^OH scavenging activity was conducted with a hydroxyl radical antioxidant capacity (HORAC) assay kit (Cell Biolabs, Inc., USA).Scavenging activity of O_2_^−^ was measured with a SOD assay kit (Sigma-Aldrich, USA).

### Toxicity evaluation

For in vitro cytotoxicity study, murine macrophage (RAW264.7) cells were cultured in Dulbecco’s Modified Eagle’s Medium (DMEM) accompanied with 10% fetal bovine serum (FBS) and 1% penicillin/streptomycin under 5% CO_2_ at 37 °C. RAW264.7 cells (10^4^/well) were seeded in 96-well plates and cultured for 24 h with 5% CO2 at 37 °C. Then, DMEM solutions of nanoparticles with different concentrations (0, 3.75, 7.5, 15, 30, 60, and 120 μg/mL) were added to the wells. After another 24 h or 48 h incubation at 37 °C under 5% CO_2_, the cell viability was measured by MTT assay.

For the in vivo toxicity study, seven-week old female Kunming mice (∼25 g) were set into three groups randomly. After the intravenous injection of Ceria@Apt at a dose of 150 μL (2 mg/kg) (n = 3), blood samples and organs were harvested from two groups at 1 day and 30 days post-injection. The mice receiving saline injection were used as the control group. For blood test, about 15 μl of blood samples were collected from the same mice at each time points. H&E-stained organs were performed to monitor the histological changes in the heart, liver, spleen, lungs, and kidneys of the mice. The complete blood panel data from the Ceria@Apt-treated and control groups were measured. The blood samples were then centrifuged at 2000 *g* for 15 min at 4 ºC to obtain the plasma for analysis of liver functional profiles.

### In vivo PET imaging

To monitor the biodistribution in vivo, 150 μL (∼0.5 mCi) of ^89^Zr-Ceria@Apt dispersed in PBS was intravenously injected into mice (n = 3). Serial PET imaging was performed within two weeks post-injection. ROI analysis of each PET image was applied to calculate the percentage of injected dose per gram of tissue (%ID/g) in main organs with vendor software (Inveon Research Workplace) on decay-corrected whole-body images. All major organs and tissues were collected after the last PET imaging at 14 days to measure the radioactivity of each tissue and organ with a gamma counter.

### Hepatic IRI repair with Ceria@Apt

For preparing hepatic IRI model [28], the Balb/C mice were anesthetized and a midline incision was made in the abdomen, followed by moving intestines towards the left side of the abdominal cavity. After exposing the portal triad, all structures in the portal triad (hepatic artery, bile duct, and portal vein) of the left and median liver lobes were blocked with a microvascular clamp for 60 min, followed with reperfusion by removing the clamp. Then, intestines were carefully moved into the peritoneal cavity and abdomen was then sealed layer by layer with sterile medical silk suture. In the sham group, the liver was exposed under the same surgical procedure except for the ligation. Liver tissues and blood were harvested for further analysis at 12 h after reperfusion.

The Ceria@Apt was mainly accumulated in the liver at 1 day post-injection according to PET imaging results. For treatment of hepatic IRI, the Ceria@Apt (0.6 mg/kg) or PBS were intravenously injected into mice (n = 4) at 1 day before the surgery. The mice in the sham group and healthy mice treated with the Ceria@Apt (0.6 mg/kg) or PBS were used as a control group (n = 4). After 12 h of the hepatic IRI model induction, their liver function was evaluated.

### Confocal imaging of superoxide production in liver tissues

To evaluate superoxide production in liver, collected liver tissues were buried in optimum cutting temperature (O.C.T.) specimen matrix for cryostat sectioning at − 20 ºC (VWR, Radnor, PA, USA). About 5 μm thickness of frozen liver tissue slices were washed with PBS and stained with dihydroethidium (DHE, 1 mM) for 0.5 h to detect superoxide formation. Subsequently, a cover glass was applied to each slide using Vectashield mounting medium (Vector Laboratories, Burlingame, CA, USA) for confocal imaging.

### Measurement of cytokines

Obtained liver tissues were cut into small pieces, and then homogenized in PIPA buffer (Boston Bio Products) containing 1 × protein inhibitor at a final concentration of 200 mg/mL. All processes were conducted on ice. after 20,000 g centrifugation for 20 min at 4 °C, lysates were obtained and stored at -80 °C until use. Before tests, samples were thawed on ice and diluted in a serial of dilutions (1:10 to 1:500). The following measurements of cytokines by using enzyme-linked immunosorbent assay (ELISA) were conducted according to the instructions from manufacturers (Mouse IL-1β ELISA development Kit, PromoKine; Mouse IL-6 ELISA Kit, Bioleagend; Mouse TNF-α ELISA Kit, Cayman; Mouse NOS2 ELISA Kit, G-Bioscience; Mouse Myeloperoxidase ELISA Kit, R&D system).

### Statistical analysis

The results were presented as the mean ± standard deviation. The statistical significance of the differences was determined by two-tailed Student’s t-test. Values with P < 0.05 were recognized as statistically significant (*means *p* < 0.05, **means *P* < 0.01, ***means *P* < 0.001).

## Results and discussion

### Synthesis and characterization of Ceria@Apt

The nanoceria were synthesized and surface modified with DSPE-PEG_5K_-NH_2_ and DSPE-PEG_5K_ according to the previous literature. [28, 47] As shown in TEM image (Fig. 1b), the synthesized nanoceria exhibited shape uniformity with 5 nm average diameter. The existence of all the expected basic chemical elements (Ce and O) was confirmed by energy-dispersive X-ray (EDX) spectrum (Additional file 1: Figures S1) and the powder X-ray diffraction (XRD, Additional file 1: Figure S2) spectrum displayed pure and typical fluorite cubic structure with high crystallinity. After PEGylation, nanoceria exhibited a positive zeta-potential (+ 31.3 mV) due to the -NH_2_ surface group, which could be easy absorbed by anti-C5a aptamer with a negative surface zeta-potential (− 12.8 mV) due to the electrostatic attraction. The final surface charge decreased to + 11.5 mV for Ceria@Apt (Additional file 1: Figure S3). Also, no significant changes ocurred in the morphology of nanoceria after the surface modification (Fig. 1c), and Ceria@Apt retained a well-defined size distribution after anti-C5a aptamer conjugation (Additional file 1: Figure S4).

As a traditional nano-antioxidant, nanoceria was reported to scavenge multiple ROS by the switch of valence state. The Ce^4+^ sites are responsible for the oxidation of H_2_O_2_ as catalase (CAT)-mimetics, and the Ce^3+^ sites are known to remove **•**OH via redox reactions and clear O_2_^−^ via superoxide dismutase (SOD)-mimetics [48–50]. In our previous study, we have confirmed a recyclable antioxidative capability in nanoceria by X-ray photoelectron spectroscopy (XPS), electron spin resonance (ESR) and Raman spectroscopy [28]. Herein, three representative ROS, H_2_O_2_, O^2−^, and •OH, were used to briefly investigate the ROS-scavenging activity of Ceria@Apt. As shown in Fig. 1d–f, Ceria@Apt remained robust multiple ROS scavenging capability that depended on cerium concentrations, showing their potential antioxidative roles in repair of hepatic IRI.

### In vivo biodistribution of Ceria@Apt

**Fig. 2:**
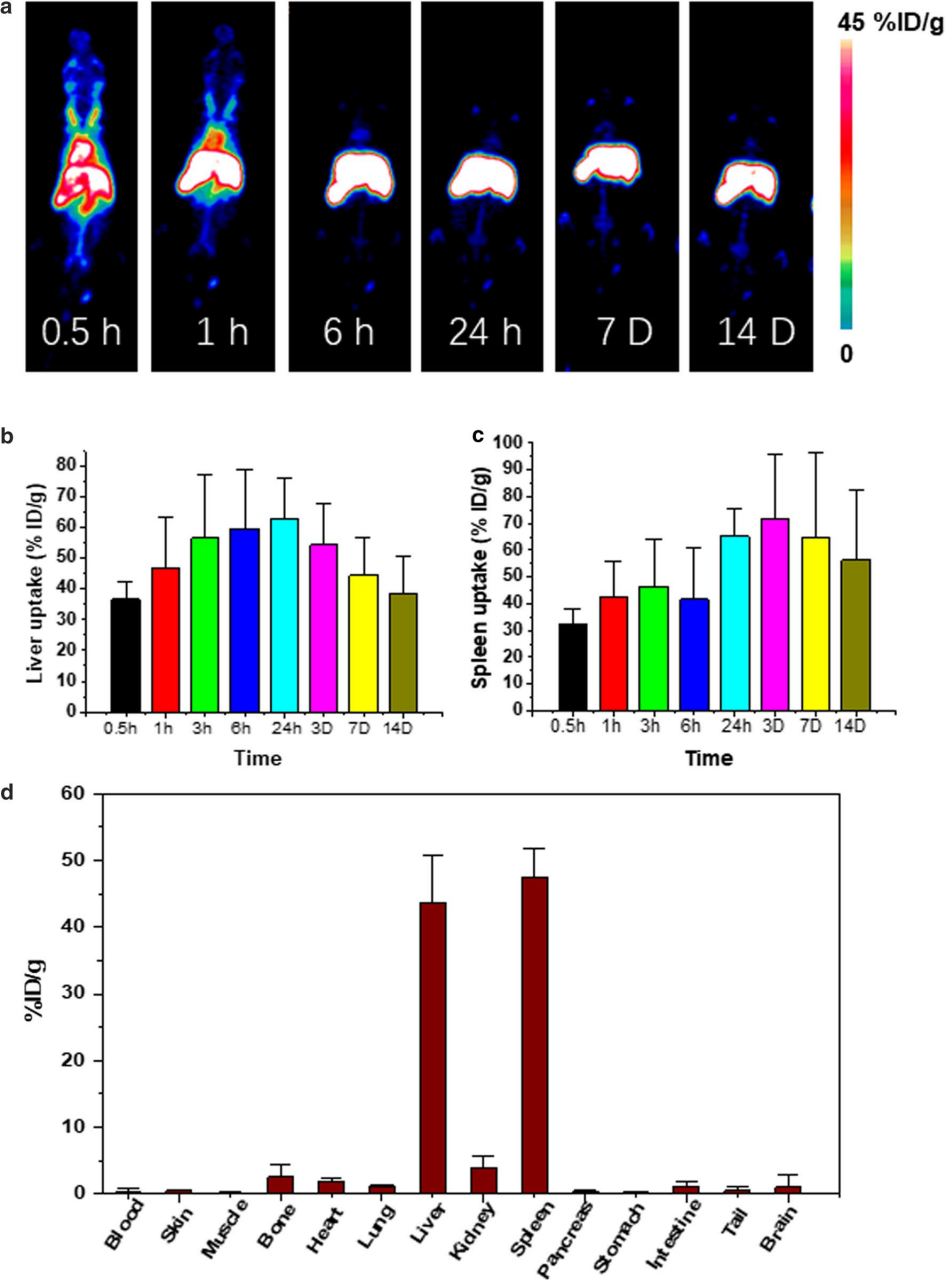
In vivo PET imaging of Ceria@Apt. **a** Representative maximum intensity projection PET images of mice at different points after intravenous injection of ^89^Zr-Ceria@Apt. Quantification of PET images for ^89^Zr-Ceria@Apt uptake in **b** liver and **c** spleen at various time points post-injection. **d** Ex vivo biodistribution of ^89^Zr-Ceria@Apt as determined by measuring ^89^Zr radioactivity in different tissues and organs (n = 3, mean ± s.d.) after the final PET image was collected

To reveal the biodistribution of Ceria@Apt via positron emission tomography (PET) imaging, Ceria@Apt was easily labelled with radionuclide ^89^Zr because Zr^4+^ was found to be easily incorporated within nanoceria, [28, 51] which was confirmed by autoradiographic image of thin layer chromatography (TLC) in Fig. 1g. The ^89^Zr labeling yield reached 94.6% at 55 °C after 2 h of incubation. More than 87% of ^89^Zr-Ceria@Apt were remained intact as monitored by TLC for up to three days (Additional file 1: Figure S5), indicating that ^89^Zr-Ceria@Apt were highly stable in both PBS and blood serum. After intravenous injection of ^89^Zr-Ceria@Apt, PET imaging was performed with two weeks post-injection. As shown in Fig. 2a, PET maximum intensity projection images, dominant liver and spleen accumulation after 1 h p.i. and was the main signal from 6 h to 14 days post-injection. High in vivo stability of ^89^Zr-Ceria@Apt over this time was indicated by negligible ^89^Zr bone and joint uptake (decorporated ^89^Zr is absorbed by the bones and joints). Then, quantitative region-of-interest (ROI) analysis of PET images was conducted and liver uptake of ^89^Zr-Ceria@Apt peaked 1 d p.i. (62.7 ± 13.3%ID/g, n = 3), which subsequently decreased over 14 d p.i. (Fig. 2b). Ex vivo biodistribution studies further confirmed the quantification data from the ROI analysis of PET images (Fig. 2d). In brief, sustained Ceria@Apt accumulation in the liver indicated a continued hepatic IRI repair potential in vivo.

### In vivo cytotoxicity of Ceria@Apt

**Fig. 3:**
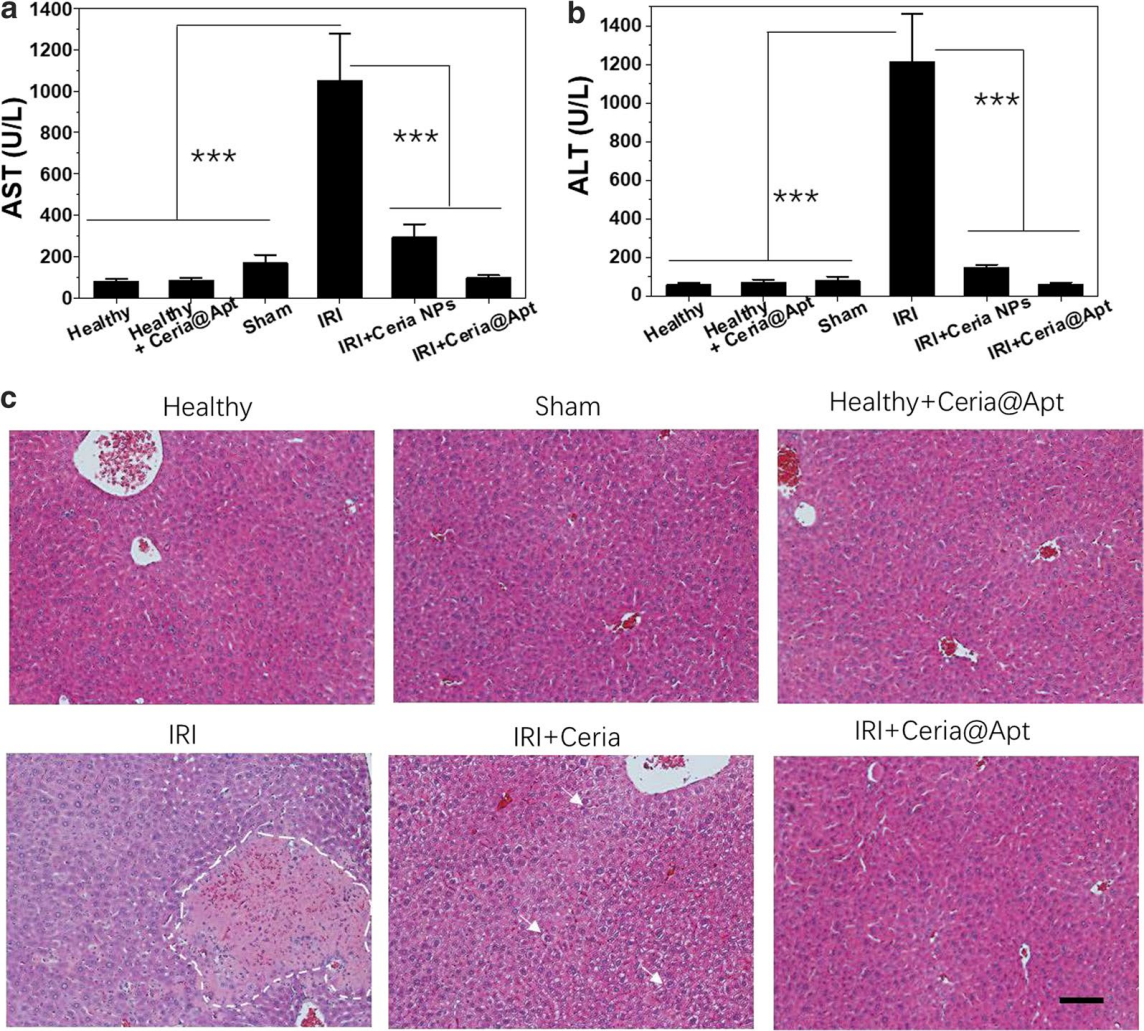
In vivo hepatic IRI repair of Ceria@Apt. **a** AST and **b** ALT levels in blood serum from hepatic IRI mice received different treatments. (n = 4, mean ± s.d.; *P* values were calculated by two-tailed Student’s *t*-test, ***P < 0.001); **c** H&E staining of liver tissues from hepatic IRI mice receiving different treatments. White dash line indicates the severely damaged tissue areas, while the white arrows indicate the formation of lipid droplets. Scale bar: 100 μm

Firstly, no obvious cytotoxicity of Ceria@Apt against murine macrophage (RAW264.7) cells after co-incubating for 24 h or 48 h was found (Additional file 1: Figure S6). Potential in vivo toxicity of Ceria@Apt was then assessed before the in vivo application for hepatic repair. All mice were randomly divided into two groups and intravenously injected with Ceria@Apt to evaluate short-term toxicity (lasting 1 day) and long-term toxicity (lasting one month). Another group was received PBS injection as control group. The standard blood parameters were measured. Meanwhile, the weights of all mice were recorded every two days and the main organs (heart, liver, spleen, lung, and kidney) of all groups were collected after corresponding days for hematoxylin–eosin staining (H&E) staining. As shown in Additional file 1: Figure S7–10, for the hematology analysis, the blood parameters in the Ceria@Apt-treated groups both in short and long term appeared to be normal compared with the control group, and no obvious in vivo toxicity of main organs was found for mice injected with the Ceria@Apt (Additional file 1: Figure S11, Fig. 3).

### Hepatic IRI repair with Ceria@Apt

After confirming the liver accumulation of Ceria@Apt by the PET imaging and negligible toxicity in vivo above, the capability of Ceria@Apt for hepatic repair was evaluated. The murine model of hepatic IRI was successfully established by a previously reported method. The repair effect of Ceria@Apt for hepatic IRI was compared between different groups, including PBS-injected IRI group, nanoceria-injected IRI group, and Ceria@Apt injected IRI group. Also, the sham surgical procedure without ligation of the portal triad was tested in healthy mice. Two clinical widely used indicators, aspartate aminotransferase (AST) and alanine aminotransferase (ALT) levels were measured for monitoring liver health 12 h post operation. The blood samples/liver from all groups were also collected for further evaluation of H&E staining, inflammatory cytokine measurement, and biomarkers detection.

**Fig. 4:**
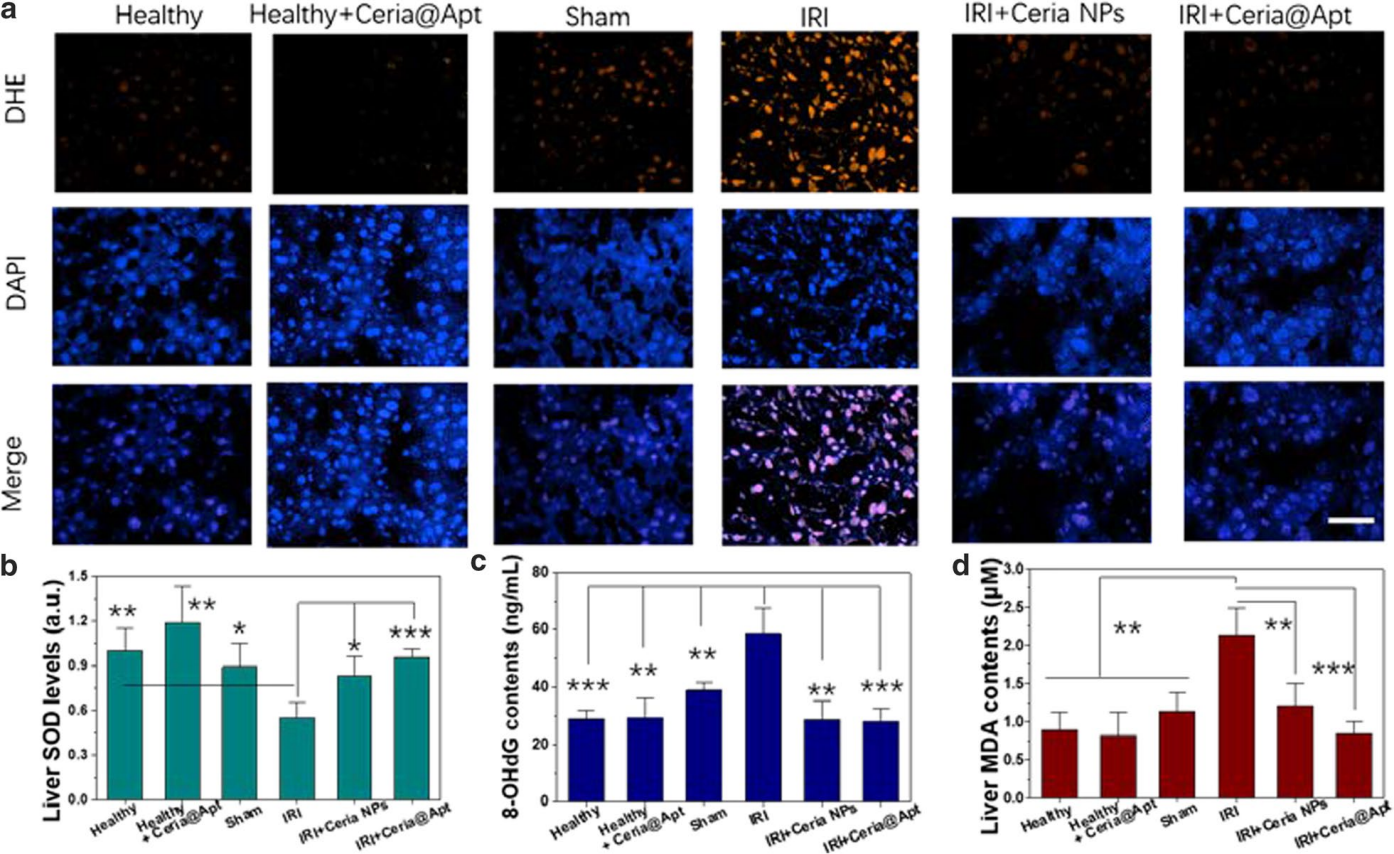
Biomarkers of liver tissue after treatments. **a** DHE and DAPI staining of liver tissues from each group. Scale bar: 50 μm. Levels of **b** SOD, **c** 8-OhdG, and **d** MDA in liver tissue homogenates from each group. (n = 4, mean ± s.d.; *P* values were calculated by two-tailed Student’s *t*-test, *P < 0.05; **P < 0.01; ***P < 0.001)

**Fig. 5:**
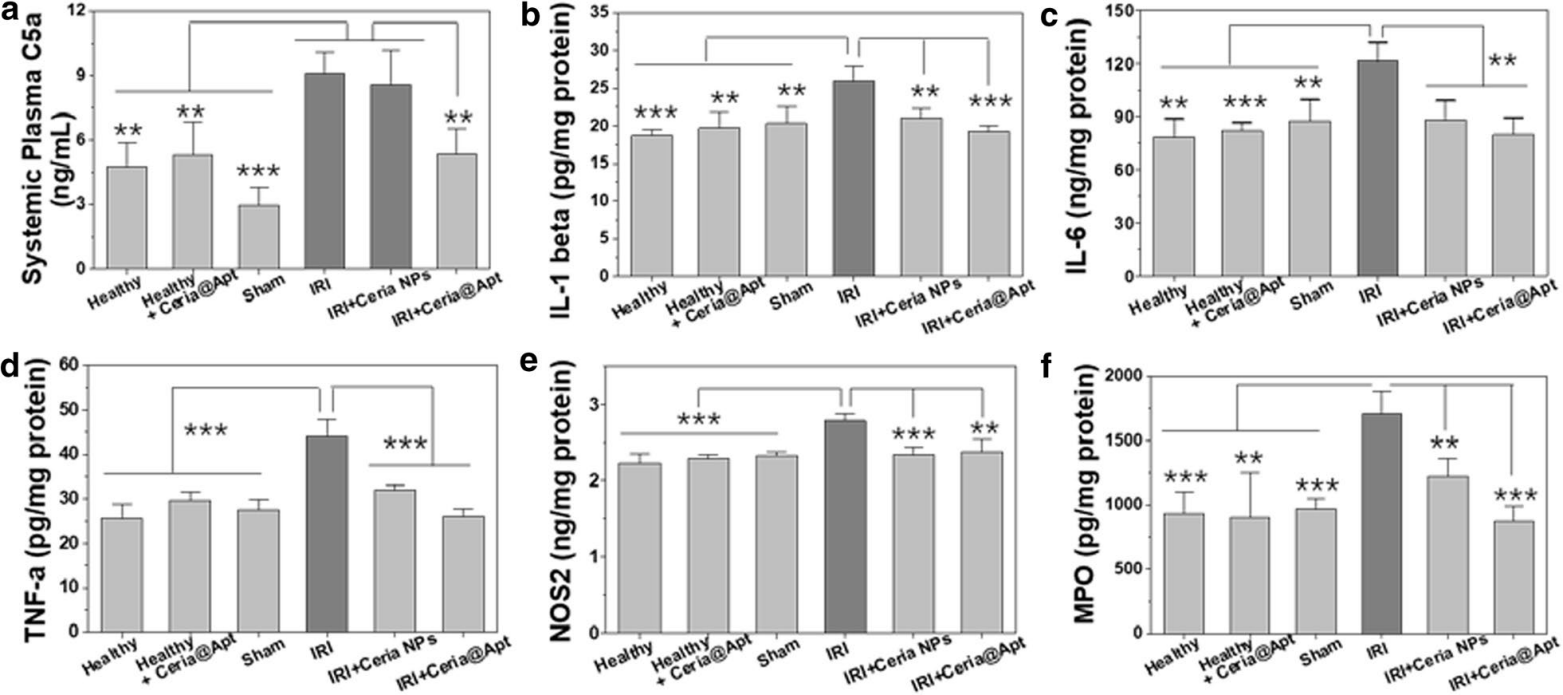
Detection of cytokines in liver tissues. The change of **a** C5a in plasma from different groups. Cytokines of **b** IL-1, **c** IL-6, and **d** TNF-α measured in liver homogenates form each group. **e** NOS2 from activated monocyte/ macrophages and Kupffer cells, and **f** MPO from activated neutrophil were measured in liver homogenates form each group. (n = 4, mean ± s.d.; *P* values were calculated by two-tailed Student’s *t*-test, *P < 0.05; **P < 0.01; ***P < 0.001)

As shown in Fig. 4a, b, compared to healthy mice, both AST and ALT levels of IRI mice increased significantly post operation. This effect was significantly reduced in the Ceria@Apt treated-IRI group, suggesting alleviated liver injury and a therapeutic effect of Ceria@Apt. A much lower value of liver indicators was found for Ceria@Apt treated group than that of nanoceria-only treated group, suggesting the feasibility of C5a-blocking strategy. A direct evidence of hepatic IRI treatment was provided by H&E staining of liver tissues, where a large area of severe damage in liver section from IRI mice receiving PBS treatment was observed (see Fig. 4c). However, only minor damage of liver tissues was found for nanoceria-treated IRI group with some structures of lipid droplets (marked as yellow arrows), indicating the anti-oxidative effect of nanoceria in preventing IRI. In contrast, no damage was found in the Ceria@Apt treatment group, which showed histological features similar to healthy mice that received PBS or Ceria@Apt injection. To further confirm the C5a-blocking method in hepatic IRI repair, C5a was measured from excised liver tissues, and higher concentrations of C5a were found in PBS-treated IRI mice compared to those in the healthy and sham groups (Fig. 5a). Decreased levels of C5a were found after Ceria@Apt treatment, suggesting that the increased C5a after hepatic IRI could be attenuated with Ceria@Apt treatment. All these results demonstrated the successful hepatic IRI repair by the proposed antioxidant and C5a blocking strategy.

### Biomarkers and cytokines detection in liver tissues

Dihydroethidium (DHE) staining was used to evaluate the production of superoxide anion in liver tissues. As shown in Fig. 4a, the abundant superoxide anion was generated in liver tissues from hepatic IRI mice received PBS injection, which was inhibited by the treatment with Ceria@Apt. Superoxide dismutase (SOD) is reported to neutralize ROS for hepatic cells, which has been a biomarker of oxidative stress. As shown in Fig. 4b, hepatic SOD levels in Ceria@Apt treated-IRI mice were recovered to similar levels of healthy mice, while significantly reduced levels of SOD were found in IRI mice with PBS treatment. In addition, DNA oxidative damage and lipid peroxidation during hepatic IRI was assessed by measuring liver 8-hydroxy-2′-deoxyguanosine (8-OHdG) and malondialdehyde (MDA) levels. Levels of 8-OhdG and MDA in liver tissues from PBS-treated IRI mice were increased in comparison with those healthy mice (Fig. 4c, d), but both were reversed into normal range in group of Ceria@Apt treated IRI mice, suggesting alleviated DNA damage and lipid peroxidation by Ceria@Apt treatment.

Liver tissues from all groups were collected and homogenized to measure several related pro-inflammatory cytokines, including interleukin-1 (IL-1), interleukin-6 (IL-6), and tumor necrosis factor-a (TNF-α). As shown in Fig. 5b–d, the levels of these cytokines were increased for hepatic IRI but decreased to normal ranges in IRI mice receiving Ceria@Apt injection. Nitric oxide synthase 2 (NOS2), another pro-inflammatory cytokine that produces NO at sustained high levels, [52] was ameliorated in Ceria@Apt injected-IRI mice compared to the IRI mice that received PBS injection (Fig. 5e). We suggest therefore that the overproduction of the highly toxic peroxynitrite anion in combination with additional superoxide anion was inhibited by Ceria@Apt. Meanwhile, the level of myeloperoxidase (MPO) in livers from mice undergoing hepatic IRI was reduced to normal range in Ceria@APT treated mice, suggesting inhibition of neutrophils recruitment (Fig. 5f). All these results indicated the release of pro-inflammatory cytokine and the recruitment of neutrophils of hepatic IRI mice was restricted after treatment. To summarize, ROS scavenging, C5a-blocking, reduced recruitment of neutrophils and released pro-inflammatory cytokines by Ceria@Apt treatment for hepatic IRI repair were confirmed in a small animal model.

## Conclusion

In summary, by utilizing the liver uptake effect of nanomaterials, a dual strategy of antioxidant and C5a-blocking by using Ceria@Apt was proposed for hepatic IRI repair in living animal. Importantly, the detailed in vivo process of Ceria@Apt in repairing hepatic IRI has been investigated, including ROS scavenging, C5a-blocking, DNA damage/lipid peroxidation, and decreased release of pro-inflammatory cytokines. All results confirmed the Ceria@Apt’s effects on hepatic liver repair with preferred liver uptake of nanomaterials. However, PET imaging indicated a 14 days longer accumulation of Ceria@Apt in the liver after preventing IRI, which required an in-depth investigation in future to promote their quick clearance from the body after treatment. Overall, the proposed strategy provided a foundation for future research on other different kinds of nano-antioxidants or treating other IRI caused by general surgery.

## Supplementary Information

**Additional file 1. **Additional figures.
