# Supplementary material for: Antioxidant and C5a-blocking strategy for hepatic ischemia–reperfusion injury repair
Source: J Nanobiotechnology. 2021 Apr 15;19:107. doi: 10.1186/s12951-021-00858-9 (PMC8050892; doi:10.1186/s12951-021-00858-9)
Supplement: Supplementary file 1 — Additional file 1. Additional figures. [file 12951_2021_858_MOESM1_ESM.doc]

***Supplementary Information***

Antioxidant and C5a-blocking Strategy for Hepatic Ischemia-Reperfusion Injury Repair

Xiaobing Zhang,^1,†^ Jiajia Hu,^2,†^ Kaelyn V. Becker, ^3^ Jonathan W. Engle,^3^ Dalong Ni,^3,*^ Weibo Cai,^3^ Dong Wu,^1,*^ Shuping Qu^1,*^

^1^Department of Hepatobiliary Surgery, Eastern Hepatobiliary Surgery Hospital, Second Military Medical University, Shanghai 200438, P. R. China.

^2^Department of Nuclear Medicine, Ruijin Hospital, Shanghai Jiaotong University School of Medicine, Shanghai 200025, P. R. China.

^3^Departments of Radiology and Medical Physics, University of Wisconsin–Madison, WI 53705, USA.

* Corresponding author

E-mail address: ndl12353@rjh.com.cn (D. Ni); WuDongEHBH@126.com (D. Wu); shupingqu33@sina.com (S. Qu)

^1^ X. Zhang and J. Hu contributed equally to this work.

**Supplementary Figures**


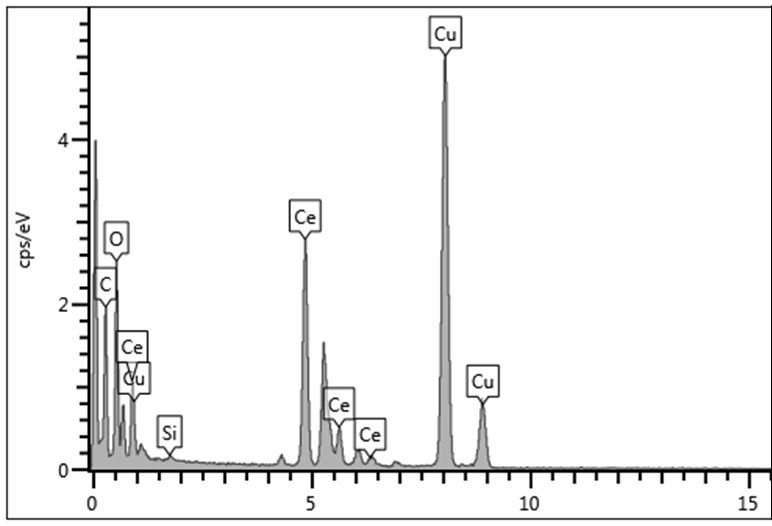


**Figure S1.** The energy dispersive X-ray (EDX) spectrum of Ceria, indicating the existence of all the essential chemical elements (Ce and O). Elements of Cu and C came from the copper grid with carbon membrane when preparing the sample.

**Figure S2.** X-ray powder diffraction of Ceria indicated a pure and typical fluorite cubic structure.

**Figure S3. The zeta-potentials test of all samples.**

**Figure S4.** The size distribution of Ceria@Apt used in this work.

**Figure S5.** The stability of ^89^Zr- Ceria@Apt in PBS and serum measured at different incubating time points (n = 3, mean ± s.d.).


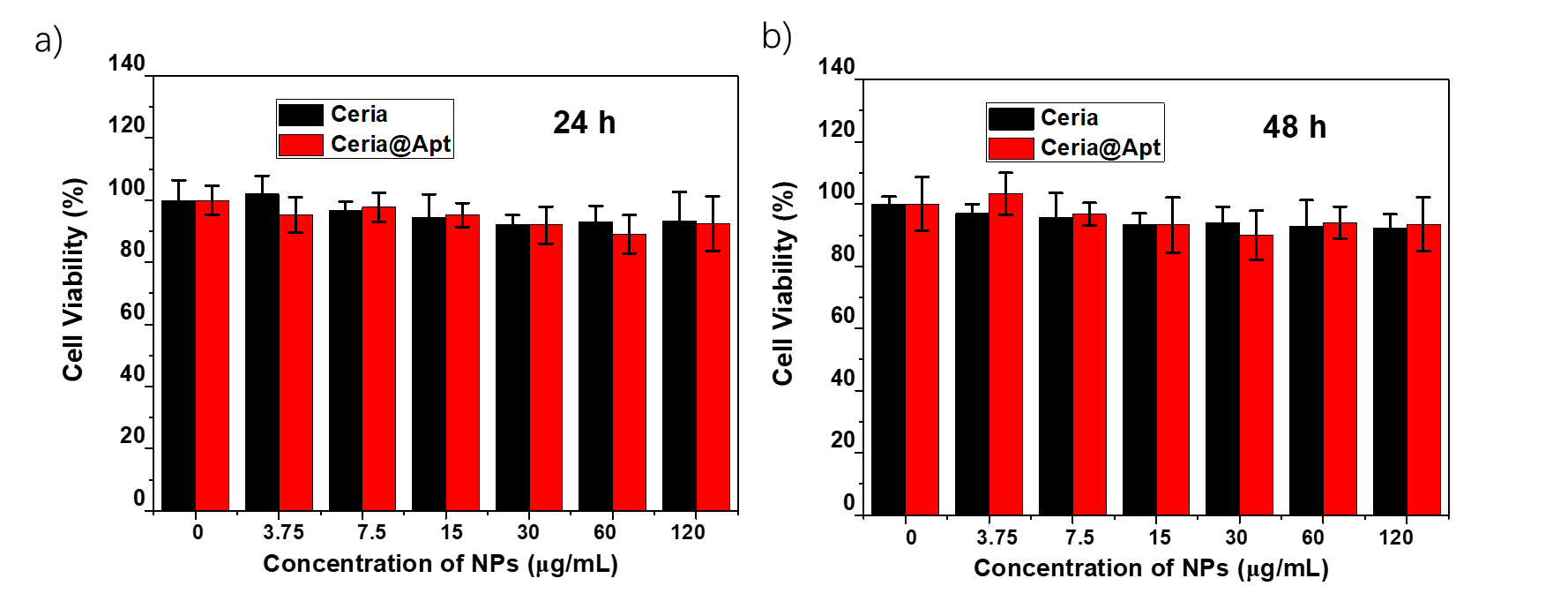


**Figure S6.** Viabilities of RAW264.7 incubated with varied concentrations of Ceria or Ceria@Apt for 24 or 48 h.

**
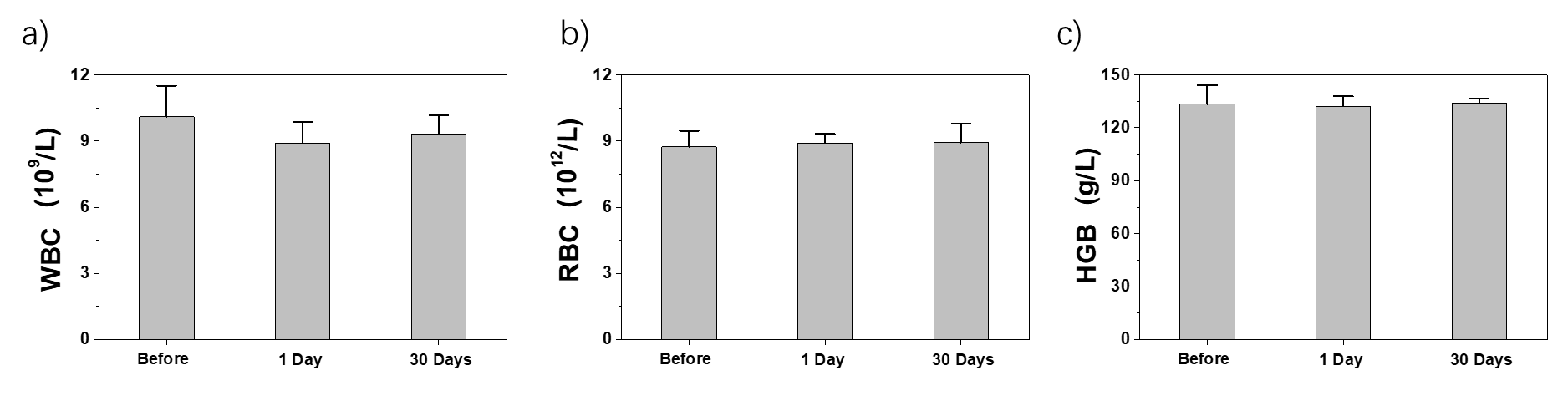
**

**Figure S7.** Numbers of a) white blood cells (WBC), b) red blood cells (RBC), and c) hemoglobin (HGB) in healthy mice before and after intravenious injection of Ceria@Apt (n = 3, mean ±s.d.).


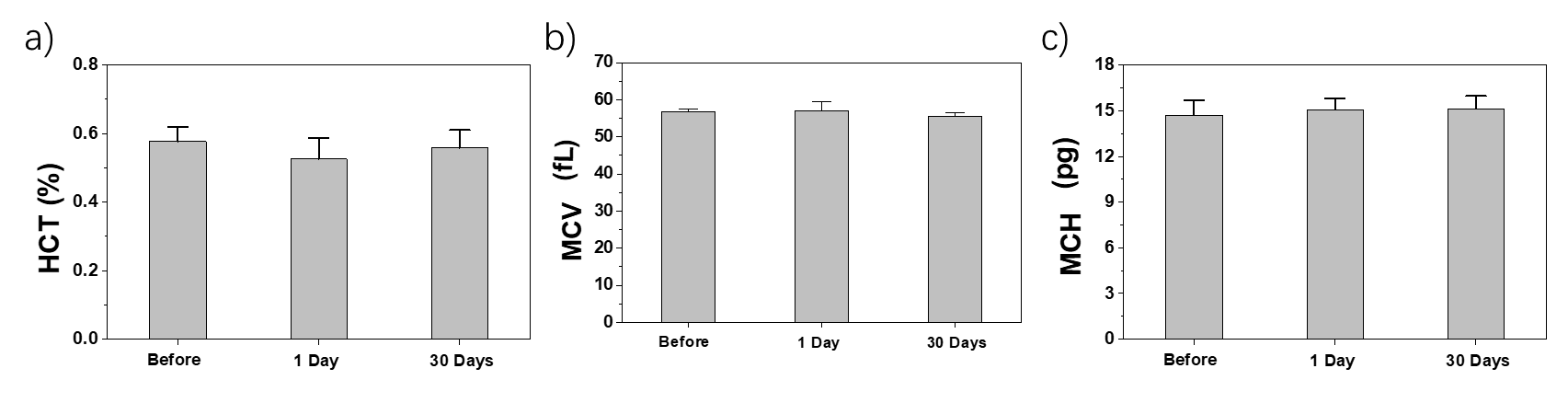


**Figure S8.** Numbers of a) hematocrit (HCT), b) mean corpuscular volume (MCV), and c) mean corpuscular hemoglobin (MCH) in healthy mice before and after intravenious injection of Ceria@Apt (n = 3, mean ±s.d.).


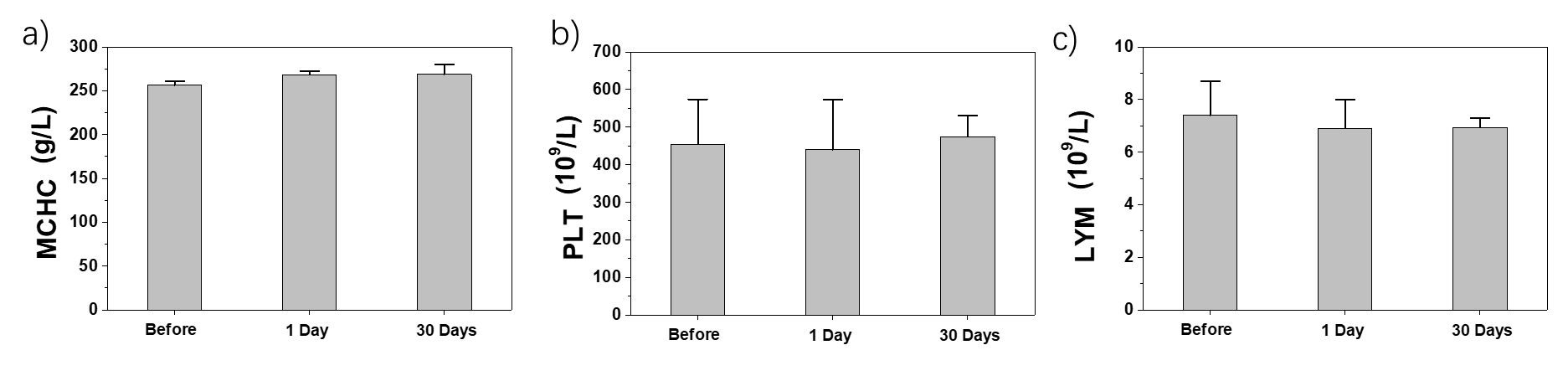


**Figure S9.** Numbers of a) mean corpuscular hemoglobin concentration (MCHC), platelets (PLT) and lymphocytes (LYM) in healthy mice before and after intravenious injection of Ceria@Apt (n = 3, mean ±s.d.).


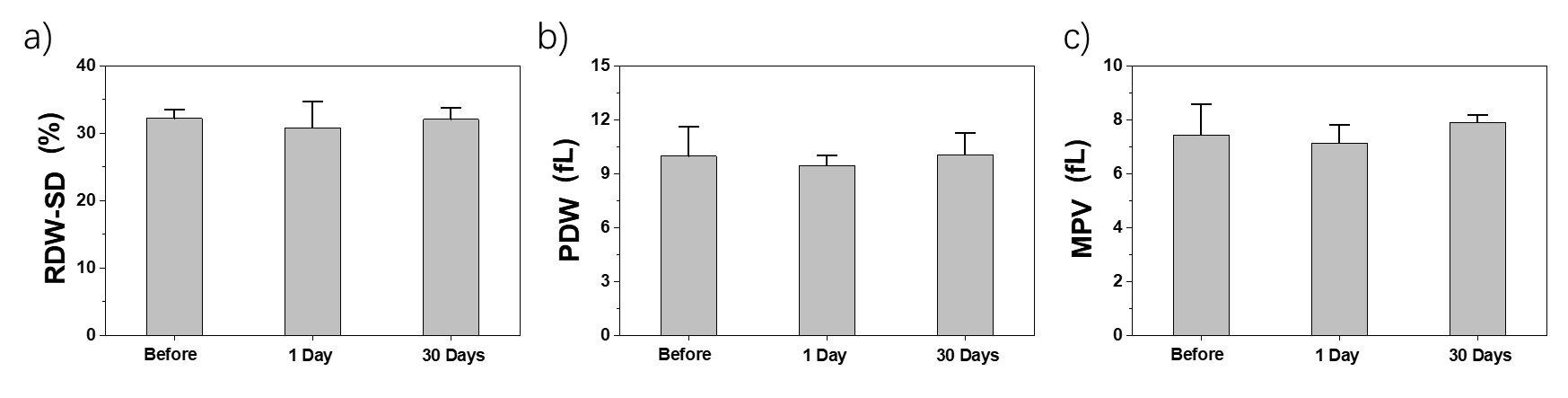


**Figure S10.** Numbers of a) red blood cell distribution width-standard deviation, (RDW-SD), platelet distribution width (PDW) and mean platelet volume (MPV) in healthy mice before and after intravenious injection of Ceria@Apt (n = 3, mean ±s.d.).

**
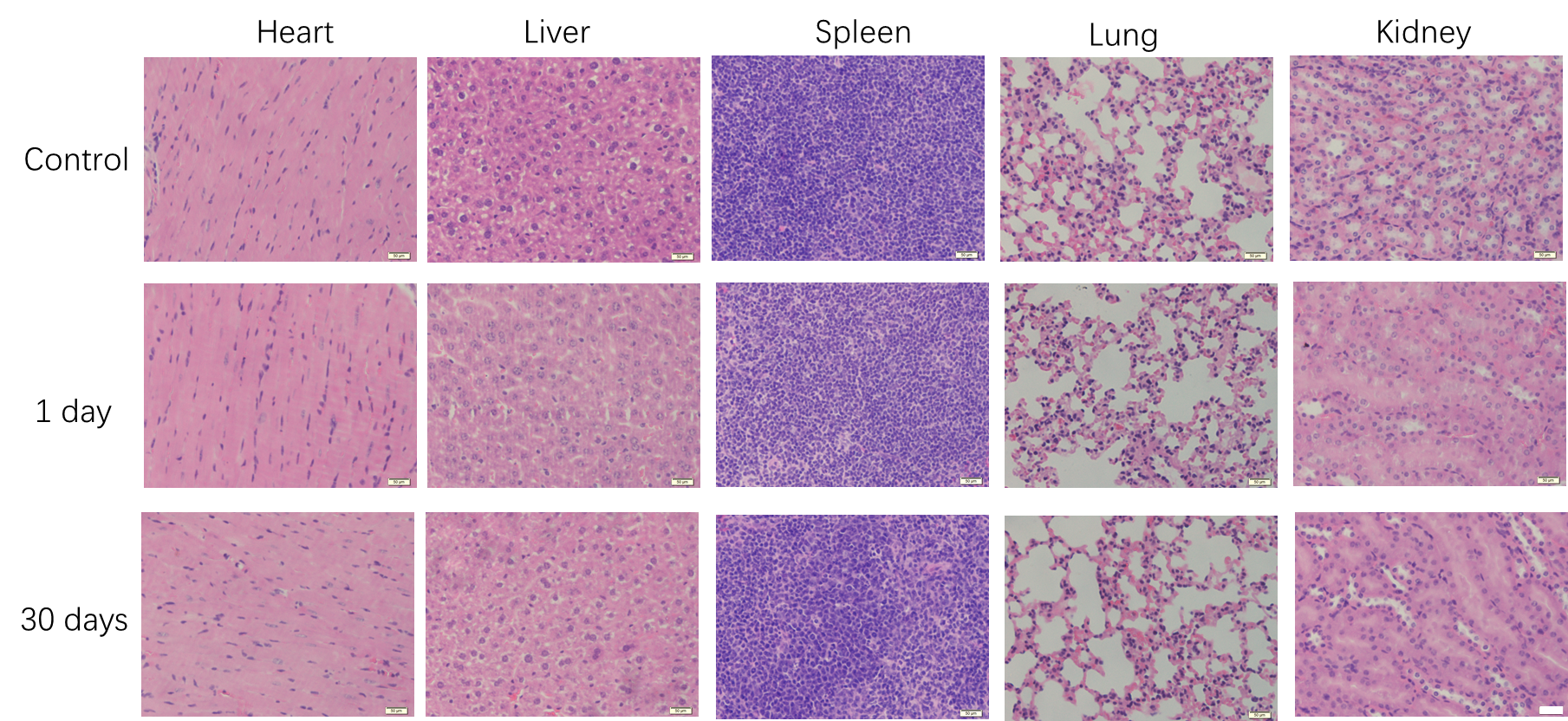
**

**Figure S11.** H&E-stained images from the heart, liver, spleen, lung, and kidney of healthy mice at 1 day or 30 days after injection of Ceria@Apt. Scale bar: 50 μm.
